# Influence of Different Dosage Forms on Pharmacokinetics of 6 Alkaloids in Raw Aconiti Kusnezoffii Radix (*Caowu*) and Chebulae Fructus- (*Hezi*-) Processed *Caowu* by UPLC-MS/MS

**DOI:** 10.1155/2020/1942849

**Published:** 2020-09-21

**Authors:** Meiru Zhi, Kaiyang Liu, Shu Han, Jinkai Xu, Weifei Li, Feng Li, Xitao Han, Yanan Tang, Ziqin Liu, Hongyue Wang, Hong Du

**Affiliations:** Beijing University of Chinese Medicine, Beijing 102488, China

## Abstract

**Purpose:**

To study the pharmacokinetics of the 6 alkaloids (aconitine, mesaconitine, hypaconitine, benzoylaconine, benzoylmesaconine, and benzoylhypaconine) in raw Aconiti Kusnezoffii Radix (*Caowu*) (RC) and Chebulae Fructus- (*Hezi*-) processed *Caowu* (HC) in the rats being, respectively, administrated with RC and HC in the dosage forms of powder and decoction and to demonstrate the mechanism of detoxification of HC.

**Methods:**

The rats were randomly divided into 4 groups and, respectively, given RC powder, HC powder, RC decoction, and HC decoction by intragastric administration. The contents of the 6 alkaloids in the plasma of the rats were detected at different time points by the UPLC-MS/MS method, and DAS 3.2.7 software was used to calculate, compare, and analyze the detected pharmacokinetic parameters.

**Results:**

Compared with those of the RC powder, the values of AUC_0‐*t*_ and *C*_max_ of the HC powder were all reduced, whereas the values of *t*_1/2*z*_ and *T*_max_ were mostly increased. Compared with those of the RC powder, the values of AUC_0‐*t*_, *C*_max_, and *t*_1/2*z*_ of the RC decoction were decreased and the value of *T*_max_ of the RC decoction was increased. Compared with those of the RC decoction, the values of AUC_0‐*t*_, *t*_1/2*z*_, and *C*_max_ of the diester diterpenoid alkaloids of the HC decoction were all increased. However, there was no marked difference between the pharmacokinetic parameters of the HC powder and the HC decoction.

**Conclusions:**

A decrease in the level of absorption and in the rate of elimination of the alkaloids can be detected when HC is administrated in the dosage form of the powder, explaining that in traditional Mongolian medicine (TMM), the purpose of using HC in the dosage forms of pills and powder is for decreasing the toxicity and prolonging the efficacy duration of HC. Decocting can greatly decrease the plasma concentration of the diester diterpenoid alkaloids in RC and increase their rate of elimination. The influence of decocting on RC is greater than that on HC, explaining the rationality of the steaming and boiling methods for processing *Caowu* and the rationality of boiling *Caowu* for a longer time beforehand in preparing an herb decoction containing *Caowu* in TCM.

## 1. Introduction

Aconiti Kusnezoffii Radix, the root of plant *Aconitum kusnezoffii* Reichb, is named *Caowu* in traditional Chinese medicine (TCM) [1]. It can be used to treat paralysis and deal with various aches and pains, including arthralgia [2–4]. *Caowu* was first recorded in *Sheng Nong's Herbal Classic* and classified as a toxic herb in the book. In TCM clinical practice, although *Caowu* is mostly used externally, it can be used internally after being processed. The earliest recording of the processing methods for *Caowu*, such as peeling, boiling in water, and decocting with honey, can be found in *Synopsis of Prescriptions of the Golden Chamber*.

In modern times, *Caowu* is a commonly used herb in both TCM and traditional Mongolian medicine (TMM). In TCM, *Caowu* is usually processed by the boiling or steaming method, which can turn the highly toxic diester diterpenoid alkaloids hydrolyzed into less toxic monoester diterpenoid alkaloids and amine-diterpenoid alkaloids and enlarge the security window of *Caowu*. In TMM, *Caowu* is usually processed with Chebulae Fructus, the fruits of *Terminalia chebula* Retz. and *Terminalia chebula* Retz, var. *tomentella* Kurt., which is called *Hezi* in TCM, but up to now, the mechanism of the detoxification of the *Hezi*-processing method remains unclear. Our previous study showed that the contents of diester diterpenoid alkaloids in *Hezi*-processed *Caowu* (HC) were slightly lower than those in raw *Caowu* (RC) but higher than those in water-soaked *Caowu* (WSC); although the contents of 3 diester diterpenoid alkaloids in both RC and HC decoctions were decreased, those in the HC decoction decreased at a lower level (Table 1), explaining that decocting could accelerate the hydrolysis of diester diterpenoid alkaloids whereas the *Hezi*-processing method could prevent the loss and hydrolysis of the 3 diester diterpenoid alkaloids to a certain degree. However, the acute toxicity of the HC powder was found to be significantly smaller than that of the RC powder although the contents of the 3 diester diterpenoid alkaloids in the RC powder were slightly higher than those in the HC powder [5–8]. Therefore, we speculated that the mechanism of detoxification of HC may be related to the in vivo absorption of the alkaloids.

**Figure 1:**
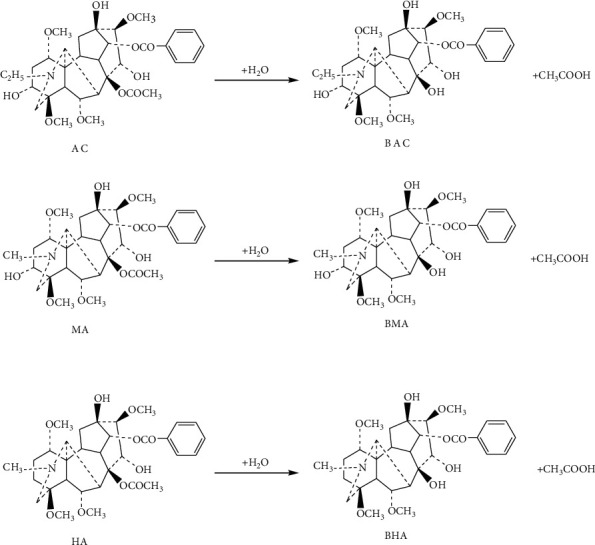
The reactions of diester diterpenoid alkaloids being hydrolyzed to monoester diterpenoid alkaloids. Notes: AC: aconitine; MA: mesaconitine; HA: hypaconitine; BAC: benzoylaconine; BMA: benzoylmesaconine; BHA: benzoylhypaconine.

Compared with the synthetic medicines, the chemical components of a single TCM herb or a group of herbs in a formula are usually complicated, and they can chemically interact with each other under certain conditions. In vitro, when an herb (or herbs in a formula) is being decocted or processed by a certain method, complicated chemical interactions of these components may occur, and in vivo, such interaction of the components will affect the absorption, distribution, and metabolism of these components [9]. In the present study, we plan to explore the mechanism of detoxification of the HC and the influence of different dosage forms on the toxicity of *Caowu*, by studying the pharmacokinetics of the 3 diester diterpenoid alkaloids, namely, aconitine (AC), mesaconitine (MA), and hypaconitine (HA), and the 3 monoester diterpenoid alkaloids, namely, benzoylaconine (BAC), benzoylmesaconine (BMA), and benzoylhypaconine (BHA), which are the 6 main bioactive components in *Caowu* [10–12] (Figure 1). As *Caowu* is mainly used in decoction in TCM and in powder and pills in TMM, RC powder (RCP), HC powder (HCP), RC decoction (RCD), and HC decoction (HCD) were chosen as the samples in our present study for detecting the pharmacokinetic parameters of the 6 alkaloids in *Caowu*.

## 2. Experimental

### 2.1. Experimental Materials

#### 2.1.1. Standard Substances

The standard substances of the 6 alkaloids, AC (purity > 98.8%), MA (purity > 99.2%), HA (purity > 98.5%), BAC (purity > 99.1%), BMA (purity > 98.6%), and BHA (purity > 94%), were all obtained from the National Institutes for Food and Drug Control (Beijing, China). The internal standard (I.S.) substance, reserpine (purity > 98%), was purchased from the Shanghai Yuanye Bio-Technology Co., Ltd. (Shanghai, China).

#### 2.1.2. Reagents and Solvents

The guaranteed reagents, methanol, acetonitrile, and formic acid, and some analytical reagents were all purchased from Thermo Fisher Scientific (Waltham, MA, USA). The ultrapure water was prepared by the Milli-Q® Reference Water Purification System (Merck Millipore, Darmstadt, Germany).

#### 2.1.3. TCM Herbs

Aconiti Kusnezoffii Radix, namely, *Caowu* in TCM, was purchased from Beijing Huamiao Pharmaceutical Co., Ltd. (Beijing, China), and Chebulae Fructus, namely, *Hezi* in TCM, was purchased from Beijing Heyanling Pharmaceutical Co., Ltd. (Beijing, China), and both of them were authenticated by Prof. Jingjuan Wang from the School of Chinese Materia Medica of Beijing University of Chinese Medicine (BUCM). HC was prepared by one of our research team members.

### 2.2. Experimental Animals

Twenty-four male Sprague-Dawley (SD) rats were provided by SPF (Beijing) Biotechnology Co., Ltd. (Beijing, China). Weighing 200 ± 20 g, the rats were raised under the following conditions: humidity = 55 ± 5%, temperature = 22 ± 2°C, and light = 12/12 h. All the rats had free access to water and foodstuff and were acclimated for at least 3 days before being used in an experiment. Before attending an experiment, all the rats were fasted for 12 h, during which, they were allowed to drink water freely. All animal experiments were made according to the Regulations of Beijing Municipality on the Administration of Laboratory Animals and were authorized by the BUCM Animal Ethics Committee.

### 2.3. Experimental Methods

#### 2.3.1. UPLC-MS/MS System and the Conditions for Operation

A Waters Xevo TQ-S Triple Quad Mass Spectrometer was connected to an ACQUITY™ UPLC system (Waters Corp., Milford, MA, USA) via an electrospray ionization source (ESI) interface. Gradient elution was performed on a Waters ACQUITY™ UPLC BEH C_18_ column (100 mm × 2.1 mm, 1.7 *μ*m) at 40°C. The mobile phase was comprised of acetonitrile and 0.1% formic acid water, and the flow rate was 0.3 mL/min. The changes in the gradient were as follows: acetonitrile was kept at 5% for 0.5 min, acetonitrile was increased from 5% to 45% for 0.5-1.5 min, acetonitrile was raised to 47% from 45% for 1.5-4.5 min, acetonitrile was increased to 95% from 47% for 4.5-6.5 min and was maintained this level for 1 min, and acetonitrile was decreased to 5% for 7.5-9.5 min and was maintained at this level for 0.5 min. The injection volume was 2 *μ*L.

The quantitative detection of the alkaloids was operated via multiple-reaction monitoring (MRM) in a positive ion mode. The working parameters of mass spectrometry were as follows: the capillary voltage was 0.58 kV, the desolvation gas (nitrogen) flow was 993 L/h, the cone gas (nitrogen) flow was 1 L/h, the source temperature was 149°C, and the desolvation temperature was 498°C. All of the data were collected and processed by using the MassLynx™ NT 4.2 software (Waters Corp., Milford, MA, USA).

#### 2.3.2. Preparation of Standard Substance and Quality Control Working Solutions

A series of standard substance and I.S. (500 ng/mL) working solutions was prepared with methanol. The standard substance working solutions were prepared according to the method described in the Section 2.3.3 of the present paper, except for the methanol (10 *μ*L) which was substituted for the standard substance working solution (10 *μ*L), and the final concentrations of the standard substance working solutions in the series were 0.05, 0.1, 0.5, 1, 5, 10, 50, 100, 250, and 500 ng/mL. The quality control working solutions of various concentrations (0.1, 50, and 400 ng/mL) were also prepared according to the same method. All the prepared working solutions were kept in a refrigerator at 4°C before use.

#### 2.3.3. Preparation of the Plasma Sample

10 *μ*L of methanol, 10 *μ*L of work solution of I.S. (500 ng/mL), 400 *μ*L of acetonitrile, and 100 *μ*L of plasma sample were mixed and vortexed for 1 min in a 2 mL centrifuge tube and then centrifuged at 10,000 rpm for 15 min. The supernatant was then transferred into another 2 mL centrifuge tube and dried under nitrogen. The residue was redissolved in 100 *μ*L of methanol.

#### 2.3.4. Validation of the Experimental Methods

*(1) Selectivity*. The blank rat plasma samples, blank rat plasma samples spiked with the 6 standard substances and I.S., and the rat plasma samples after intragastric administration of RCP, HCP, RCD, and HCD for 1 h were used for evaluating selectivity.

*(2) Linearity*. In the calibration curve, *X* was established as the analyte concentration and *Y* as the ratio of the analyte peak area to the I.S. peak area, in the concentration range of 0.05-500 ng/mL.

*(3) Precision and Accuracy*. The intra- and interday precision and accuracy were detected by measuring the 6 repetitive quality control samples in 1 day for 3 successive days. Precision was expressed as the relative standard deviation (R.S.D.), and accuracy was expressed as the proportion of the mean measured concentration to the added standard concentration.

*(4) Recovery and Matrix Effect*. Peak A was obtained by adding standard substance working solutions of 6 alkaloids of 3 concentrations (0.1, 50, and 400 ng/mL) to the nonprocessed blank plasma. Peak B was achieved by adding standard substance working solutions to the protein-removed blank plasma. Peak C was achieved by only using the standard substance working solutions of 3 concentrations. Peaks A, B, and C were adjusted by I.S. and expressed as the ratio of the peak area of 6 alkaloids to the peak area of I.S. Recovery was expressed as the ratio of peak A to peak B, and matrix effect was expressed as the ratio of peak B to peak C.

*(5) Stability*. Stability of all the samples for the experiments in our present study were detected after they were stored in a refrigerator at 4°C for 12 h and were represented as the percentage of the measured concentration with the added substance concentration.

#### 2.3.5. Preparation of RC and HC Powder and Decoction

*(1) Preparation of HC*. *Hezi* was crushed into uniform pieces and soaked in water for 1 h and then decocted in water for 1 h to gain the *Hezi* decoction. Afterwards, *Caowu* was soaked in the *Hezi* decoction in a ratio of 2 : 1 (*Caowu* : *Hezi*) for 3 days to gain HC [5].

*(2) The Preparation of RC and HC Powder and Decoction*. *Preparation of RC or HC powder suspension*: RC or HC was ground into powder and then mixed with 0.5% sodium carboxymethyl cellulose solution to form a suspension, the concentration of which was 0.1 g/mL. The RC or HC powder suspension should be prepared just before the intragastric administration.

*Preparation of RC or HC decoction*: RC or HC was soaked in water for 30 min after being sliced. After soaking, the RC or HC slices were decocted in water in a ratio of 1 : 10 (RC or HC : water, *w* : *v*) for 30 min for 2 times. The filtrates of the 2 times of decocting were merged and condensed by vacuum concentration at 40°C for gaining a decoction at a concentration of 0.1 g/mL. The RC or HC decoction should be prepared just before the intragastric administration.

#### 2.3.6. Pharmacokinetic Detection

Twenty-four rats were randomly and evenly divided into 4 groups, i.e., groups of RCP, HCP, RCD, and RCD. According to the *Pharmacopoeia of the People's Republic of China*, the dosage of boiling-processed *Caowu* is 1.5-3 g/day [1]. By a human-rat conversion on the basis of the body surface area, the dosage of the boiling-processed *Caowu* for rats is found to be 0.135-0.27 g/kg/day. However, the results of our previous study proved that this dosage was too small to be used in the experiments in our present study. After doing preliminary experiments repeatedly, we finally decided to adopt the dosage of 1 g/kg/day for the rats in the 4 groups. The plasma samples (about 0.5 mL) were collected at the time points of 0.25, 0.5, 1, 1.5, 2, 3, and 4 h, and then, they were put into the heparinized centrifuge tubes for centrifuging at 4,000 rpm for 15 min, and the supernatants were stored in a refrigerator at −20°C before being used for analysis.

The mean plasma concentration-time curves were plotted by Origin Software (version 9.0). The pharmacokinetic parameters were calculated in a noncompartmental model via DAS (version 3.2.7, Mathematical Pharmacology Professional Committee of China), which was determined by the application of Akaike's Information Criterion (AIC). For each rat, the maximum plasma concentration (*C*_max_) and its corresponding time (*T*_max_) were determined by visual inspection of the profiles. The apparent terminal elimination rate constant (*λ*) was calculated by linear regression of the natural logarithms of the terminal plasma concentrations. The terminal half-life (*t*_1/2*z*_) was derived from 0.693/*λ*. The area under the curve to the last measured point (AUC_0‐*t*_) was calculated using the trapezoidal rule. The data were shown as mean ± standard deviation (S.D.). The comparisons among the 4 groups were carried out by unpaired Student's *t*-test.

## 3. Results

### 3.1. Results of Validation of the Experimental Methods

In our present study, the MS/MS parameters were optimized in order to obtain the highest response.  Table 2 showed the optimized mass transition ion pairs for quantification in the MRM mode, including precursor and product ions.

**Figure 2:**
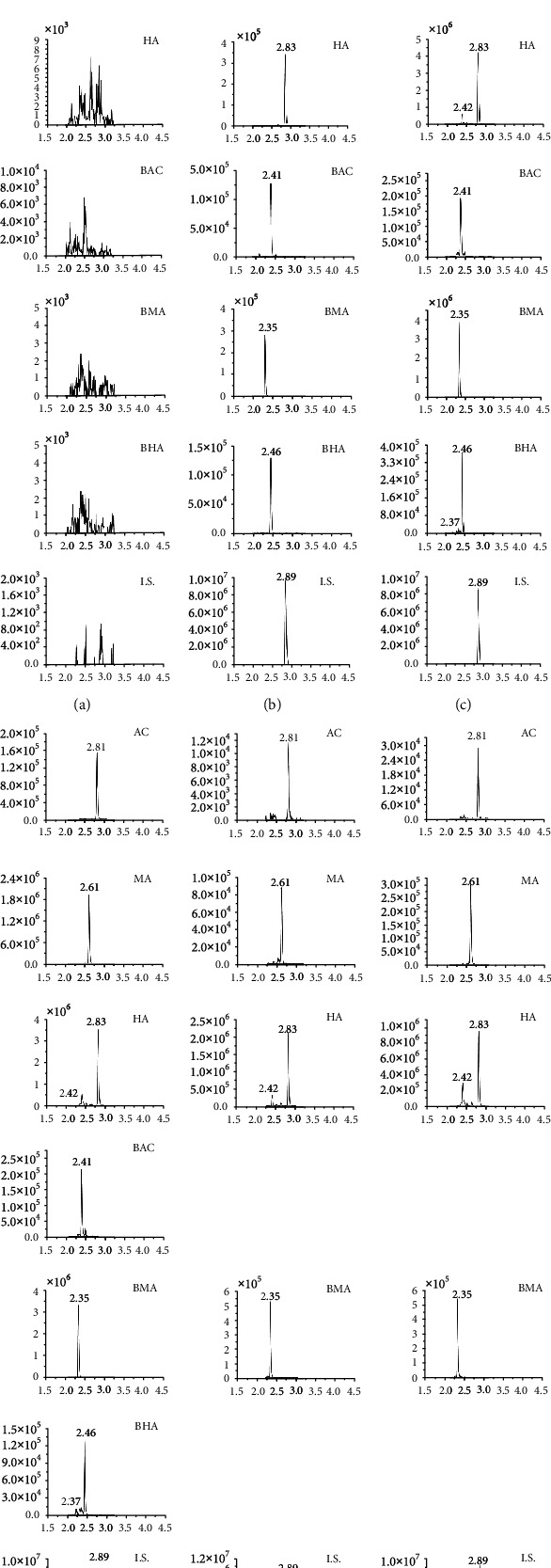
MRM chromatograms of the 6 alkaloids and I.S.: (a) blank plasma; (b) blank plasma mixed with the 6 alkaloids and I.S.; (c–f) 1 h plasma sample after administration of RCP (c), HCP (d), RCD (e), and HCD (f).

Acetonitrile, methanol, water, and formic acid, as the mobile phases, were evaluated in order to obtain their retention time and the appropriate response. It was found that compared with pure water, the 0.1% formic acid-containing water could obtain a better intensity and enhance the efficiency of ionization. Phenacetin, lappaconite hydrobromide, and reserpine, as the I.S., were evaluated, and finally, reserpine was chosen because it was found that its physicochemical properties and peak location were similar to the 6 alkaloids. The retention times of AC, MA, HA, BAC, BMC, BHC, and I.S. were 2.81, 2.61, 2.83, 2.41, 2.35, 2.46, and 2.89 min, respectively (Figure 2).

In order to remove the endogenous interference and obtain satisfactory results, different protein-removing reagents, such as methanol, acetonitrile, ethyl acetate, and diethyl ether, were tested, and finally, it was found that acetonitrile was the desired reagent.

The chromatograms in Figure 2 showed that there were no endogenous impurities interfering with the detection of the analytes and I.S. and that the specificity of the methods for detecting the analytes and I.S. could thereby be confirmed. The results of the calibration of the plasma samples are shown in Table 3, and it could be seen that the correlation coefficients (*R*^2^) for the 6 alkaloids were ≥0.99. The R.S.D. of the intra- and interday precision for all the 6 alkaloids were less than 15%, and the intra- and interday accuracies for all the 6 alkaloids were in a range of 89.45% to 104.00% (Table 4). The data of peaks A, B, and C are shown in Table 5, and it could be seen that the recoveries of the 6 alkaloids were above 82.04%, and the range of the matrix effects was from 85.00% to 100.70%, verifying that the methods used for the detection were feasible. The stability of the 6 alkaloids was found to be variable within ±15% (Table 6).

### 3.2. Results of Pharmacokinetic Detection

**Figure 3:**
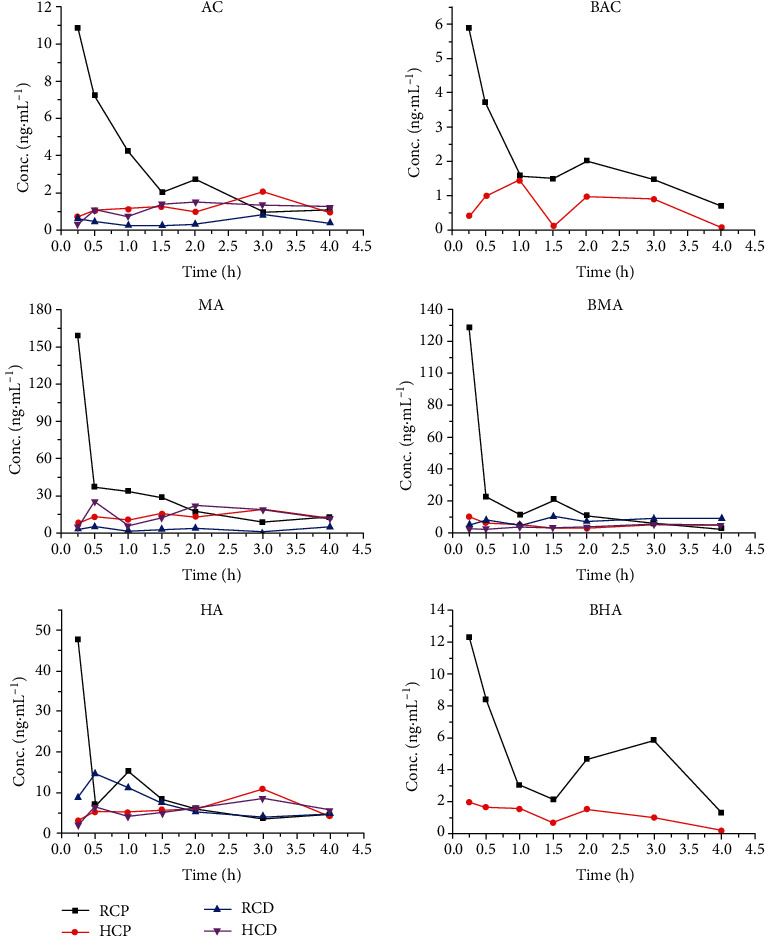
Mean plasma concentration-time curves.

The mean plasma concentration-time curves of the 6 alkaloids are shown in Figure 3, and the pharmacokinetic parameters detected after intragastric administration of RCP, HCP, RCD, and HCD are shown in Table 7. In the RCD and HCD groups, only the mean plasma concentrations and pharmacokinetic parameters of the alkaloids of AC, MA, HA, and BMA were calculated and compared, for the concentrations of the alkaloids BAC and BHA were too low to be detected.

The results of the detection showed that the mean plasma concentrations of the 6 alkaloids in HCP were significantly lower than those in RCP, and the results also showed that AUC_0‐*t*_ and *C*_max_ of HCP were lower than those of RCP, whereas *T*_max_ and *t*_1/2*z*_ of HCP were higher than those of RCP, explaining that HCP could reduce the level and rate of absorption of the 6 alkaloids, delay the rate of elimination, and prolong the time of action.

The results of the detection also showed that compared with those in RCP, the mean plasma concentrations of AC and MA were markedly lower in RCD, with a significant difference concerning AUC_0‐*t*_, *C*_max_, and *t*_1/2*z*_, implying that the level of the 2 diester diterpenoid alkaloids in plasma could be reduced and that their rate of elimination could be increased when RC was used in the dosage form of decoction.

At the same time, the results of the detection revealed that the mean plasma concentrations of AC and MA in HCD were higher than those in RCD, with a significant difference concerning AUC_0‐*t*_, *C*_max_, and *t*_1/2*z*_, whereas there was no significant difference between those parameters in HCP and HCD, implying that the influence of decocting on RC was greater than that on HC.

## 4. Discussion

In pharmacokinetics, after a medicine is administrated, the parameters AUC_0‐*t*_, *C*_max_, and *T*_max_ are used for evaluating the level and rate of its absorption, and the parameter *t*_1/2*z*_ is used for evaluating the rate of its elimination. Small values of AUC_0‐*t*_ and *C*_max_ mean a low level of its absorption and a small value of *C*_max_ and a big value of *T*_max_ mean a low rate of its absorption, whereas a small value of *t*_1/2*z*_ means a high rate of its elimination [13]. In our present study, the pharmacokinetic parameters of the 6 alkaloids in RCP, HCP, RCD, and HCD were all detected and compared in order to find the detoxification mechanism of HC.

By analysis of the results of the content detection of the 6 alkaloids in RCP and HCP (Table 1), it was found that the difference between the contents of the diester and those of the monoester diterpenoid alkaloids was little. Nevertheless, the results of the pharmacokinetic detection (Table 7) showed that the AUC_0‐*t*_ and *C*_max_ of both the diester and monoester diterpenoid alkaloids were lower and *T*_max_ was higher in HCP, compared with RCP, suggesting that the *Hezi*-processing method could cause a decrease in the level and rate of absorption of all kinds of alkaloids in *Caowu* which may be the mechanism of detoxification of the *Hezi*-processing method. The results of the pharmacokinetic detection also showed that the *t*_1/2*z*_ of most alkaloids in HCP was higher than that in RCP, implying that the *Hezi*-processing method for *Caowu* could also decrease the rate of elimination of the alkaloids in HCP and prolong their duration of action. In TMM, HC was frequently used in the dosage forms of pills and powder usually in a small dose. The results of our present study could verify that when HC was administrated in the dosage form of powder or pills, it would be much safer and exert a desirable effect in a relatively small dose.

By comparing the results of the content detection of the 6 alkaloids in RCD and RCP (Table 1), it was found that the contents of the diester diterpenoid alkaloids markedly decreased and the contents of the monoester diterpenoid alkaloids markedly increased in RCD. The results of the pharmacokinetic detection after being administrated with RCD and RCP (Table 7) showed that in RCD, the values of AUC_0‐*t*_ and *C*_max_ of the diester diterpenoid alkaloids decreased, the value of *T*_max_ increased, and some monoester diterpenoid alkaloids (such as BAC and BHA) were unable to be detected, suggesting that these monoester diterpenoid alkaloids may have undergone a further hydrolysis. The results implied that decocting could accelerate hydrolysis of all the alkaloids in RC and thereby decrease the concentrations of these alkaloids in the plasma, which contributed to the detoxification of RC. It could also be seen, from the results of pharmacokinetic detection (Table 7), that the value of *t*_1/2*z*_ significantly decreased in RCD, suggesting that decocting could increase the rate of elimination of diester diterpenoid alkaloids in RC, which also contributed to the detoxification of RC. The above-mentioned results of our present study may demonstrate the rationality of the various decocting methods used for processing RC and preparing *Caowu*-containing herb decoctions in TCM.

The results of the analysis of the contents of alkaloids in various *Caowu* samples (Table 1) showed that the contents of diester diterpenoid alkaloids in HCD were lower than those in HCP, and the contents of monoester diterpenoid alkaloids in HCD were higher than those in HCP, which was similar to the change in the contents of the various alkaloids in RCD and RCP; nothing but the level of the change was smaller, suggesting that there was a restriction of the hydrolysis of the alkaloids in *Caowu* during the processing of HC [14]. The results of the detection of the pharmacokinetic parameters (Table 7) showed that there was no marked difference between the values of the pharmacokinetic parameters of the diester diterpenoid alkaloids in HCD and HCP. However, compared with those in RCP, the values of AUC_0‐*t*_ and *C*_max_ in HCP and HCD were decreased while *T*_max_ and *t*_1/2*z*_ were increased, suggesting that in HCP and HCD, the level and rate of absorption of the diester diterpenoid alkaloids were reduced and the process of elimination of these alkaloids was prolonged [15]. It had to be pointed out that the decrease of the value of AUC_0‐*t*_ of the diester diterpenoid alkaloids in HCD may be due to the low concentration of the alkaloids in the HCD sample, whereas that in HCP may be due to the restriction of the absorption of the diester diterpenoid alkaloids caused by *Hezi*. The diester diterpenoid alkaloids in HCP could be gradually transformed into monoester diterpenoid alkaloids during a slow hydrolysis in vivo, causing the contents of the monoester diterpenoid alkaloids to increase to a detectable level. However, the free monoester diterpenoid alkaloids in HCD may be rapidly hydrolyzed in vivo, and no more monoester diterpenoid alkaloids could be supplemented by hydrolysis of the diester diterpenoid alkaloids because their contents were very low in the sample, which could explain why no BAC and BHA could be detected in HCD.

## 5. Conclusion

From the above-mentioned results of our present study, it can be concluded that the alkaloids in RCP can be absorbed at a high level and a high rate, suggesting that RCP is clinically unsafe and RC is unsuitable for being used in the dosage form of powder; that the alkaloids in HCP can be absorbed at a lower level and eliminated at a lower rate, suggesting that HCP is clinically safe and HC is suitable for being used in the dosage form of powder in a smaller dose; and that decocting can make the alkaloids in RC rapidly hydrolyzed and thereby greatly decrease the level of their plasma concentration and the intensity of toxicity of RC. The results of our present study can also demonstrate the rationality of *Caowu* being used mostly in the dosage forms of pills and powder in TMM and mostly in the dosage form of decoction in TCM.

## Figures and Tables

**Table 1:** The contents of the 6 alkaloids in different *Caowu* samples.

Kinds of alkaloids	RC (%)	HC (%)	WSC (%)	RCD (%)	HCD (%)
AC	0.363	0.384	0.227	0.060	0.105
MA	0.493	0.302	0.328	0.000	0.025
HA	0.019	0.007	0.010	0.000	0.001
BAC	0.001	0.004	0.004	0.022	0.011
BMA	0.086	0.100	0.069	0.410	0.247
BHA	0.001	0.006	0.004	0.026	0.008
Total amount of DDA	0.875	0.693	0.565	0.060	0.131
Total amount of MDA	0.088	0.11	0.077	0.458	0.266

Notes: DDA: diester diterpenoid alkaloids; MDA: monoester diterpenoid alkaloids.

**Table 2:** Mass spectrometry selection channel and related parameters.

Detecting items	Precursor ion	Product ion	Cone volt.	Col. energy
AC	646.3	586.3	14.0	32.0
MA	632.3	572.4	14.0	30.0
HA	616.4	105.0	18.0	58.0
BAC	604.4	105.0	20.0	56.0
BMA	590.3	105.0	52.0	62.0
BHA	574.3	105.0	58.0	62.0
I.S.	609.5	195.1	86.0	64.0

**Table 3:** Regression data for the 6 alkaloids.

Alkaloids	Calibration	*R* ^2^	Linear range (ng/mL)
AC	*Y* = 0.0879452*X* + 0.0532361	0.996	0.05~500
MA	*Y* = 0.119817*X* + 0.0288623	0.992	0.05~500
HA	*Y* = 0.10836*X* + 0.223721	0.994	0.05~500
BAC	*Y* = 0.0777178*X* + 0.059877	0.993	0.05~500
BMA	*Y* = 0.0663272*X* + 0.580478	0.991	0.05~500
BHA	*Y* = 0.0402479*X* + 0.116155	0.991	0.05~500

**Table 4:** Precision and accuracy of the 6 alkaloids (intraday: *n* = 6; interday: 3 days).

Alkaloids	Concentration (ng/mL)	Intraday		Interday	
		Accuracy (%)	Precision (R.S.D., %)	Accuracy (%)	Precision (R.S.D., %)
AC	0.1	94.00	12.78	95.00	4.10
	50	91.04	4.51	91.17	1.41
	400	93.79	6.34	95.20	1.30

MA	0.1	99.00	10.2	97.00	1.67
	50	90.75	5.67	89.60	1.74
	400	89.82	5.47	93.61	3.65

HA	0.1	98.00	11.12	102.00	4.84
	50	91.55	5.78	93.18	2.54
	400	89.45	4.60	92.91	4.07

BAC	0.1	90.00	14.23	93.00	5.30
	50	95.39	6.56	94.42	0.90
	400	98.29	7.49	97.30	2.42

BMA	0.1	103.00	11.89	96.00	7.93
	50	96.51	7.93	93.01	6.80
	400	98.06	6.60	99.68	1.70

BHA	0.1	104.00	11.92	95.00	8.98
	50	93.24	10.14	95.02	1.74
	400	95.22	5.39	97.24	1.81

**Table 5:** The data of peaks A, B, and C.

Compound	Concentration (ng/mL)	A	B	C
AC	0.1	0.061 ± 0.001	0.063 ± 0.001	0.065 ± 0.003
	50	4.122 ± 0.169	4.957 ± 0.181	5.845 ± 0.386
	400	33.734 ± 1.847	35.279 ± 1.727	38.799 ± 4.024

MA	0.1	0.041 ± 0.001	0.042 ± 0.002	0.043 ± 0.003
	50	5.465 ± 0.308	6.316 ± 0.393	6.986 ± 0.871
	400	43.075 ± 2.356	52.626 ± 2.185	59.124 ± 5.443

HA	0.1	0.234 ± 0.001	0.237 ± 0.001	0.239 ± 0.003
	50	5.184 ± 0.287	6.0445 ± 0.385	7.046 ± 1.194
	400	38.996 ± 1.785	47.690 ± 3.695	56.177 ± 10.792

BAC	0.1	0.068 ± 0.001	0.068 ± 0.001	0.069 ± 0.002
	50	3.704 ± 0.257	3.829 ± 0.245	4.176 ± 0.537
	400	30.836 ± 1.972	31.311 ± 2.148	33.730 ± 4.377

BMA	0.1	0.587 ± 0.001	0.587 ± 0.001	0.590 ± 0.002
	50	3.423 ± 0.233	3.946 ± 0.212	4.271 ± 0.572
	400	26.987 ± 1.55	28.085 ± 1.449	29.574 ± 2.380

BHA	0.1	0.120 ± 0.001	0.120 ± 0.000	0.119 ± 0.001
	50	1.993 ± 0.190	2.146 ± 0.144	2.166 ± 0.259
	400	15.445 ± 0.825	16.150 ± 0.402	16.441 ± 0.525

**Table 6:** Recoveries, matrix effect, and stability of the 6 alkaloids (*n* = 6).

Alkaloids	Concentration (ng/mL)	Extraction recovery (%)	R.S.D. (%)	Matrix effect (%)	R.S.D. (%)	Stability (12 h at 4°C, %)	R.S.D. (%)
AC	0.1	97.11	1.57	97.65	2.64	95.83	14.42
	50	83.31	7.22	85.00	4.87	93.49	4.40
	400	95.75	6.30	91.42	6.54	88.65	7.54

MA	0.1	97.76	5.63	96.91	4.99	97.17	8.93
	50	86.77	7.48	89.80	9.13	88.66	5.70
	400	82.04	8.27	89.36	5.37	95.54	6.49

HA	0.1	99.09	0.63	98.94	0.75	96.33	14.06
	50	86.07	8.92	87.24	11.96	91.58	9.56
	400	82.20	9.43	86.40	11.18	96.15	7.48

BAC	0.1	99.54	2.58	98.54	2.06	89.83	10.89
	50	97.20	10.67	92.49	8.79	94.91	10.03
	400	98.79	8.00	93.48	6.76	94.14	4.31

BMA	0.1	99.77	0.18	99.80	0.20	100.50	11.24
	50	87.16	11.59	93.33	9.46	97.10	8.89
	400	96.46	10.05	95.21	5.20	98.80	6.86

BHA	0.1	100.42	0.70	100.70	0.63	108.00	12.17
	50	96.35	12.10	99.74	7.09	98.07	9.28
	400	98.74	3.44	98.25	1.65	97.91	1.71

**Table 7:** Pharmacokinetic parameters of the 6 alkaloids in plasma (*n* = 6).

Alkaloids	Groups	AUC_0‐*t*_ (ng/h/mL)	*t*_1/2*z*_ (h)	*T*_max_ (h)	*C*_max_ (ng/mL)
AC	RCP	10.31 ± 3.56	2.29 ± 0.70	0.50 ± 0.00	7.23 ± 3.24
	HCP	4.96 ± 1.72^∗^^##^	2.95 ± 1.89^##^	2.38 ± 0.69^∗∗^	2.31 ± 1.16^∗∗^^#^
	RCD	1.75 ± 0.04^∗∗^	0.88 ± 0.00^∗∗^	3.00 ± 0.00^∗∗^	0.83 ± 0.19^∗∗^
	HCD	3.88 ± 0.23^∗^^##^	2.73 ± 1.19^##^	2.50 ± 0.71^∗∗^	1.60 ± 0.35^∗∗^^#^

MA	RCP	77.12 ± 1.73	0.90 ± 0.06	0.50 ± 0.00	37.03 ± 1.35
	HCP	46.90 ± 16.99^∗^^##^	2.78 ± 1.59^∗^^##^	2.17 ± 0.68^∗∗^	19.83 ± 9.03^∗∗^^#^
	RCD	10.23 ± 3.22^∗∗^	0.30 ± 0.03^∗∗^	2.25 ± 0.47^∗∗^	5.89 ± 2.22^∗∗^
	HCD	61.86 ± 3.92^##^	2.56 ± 1.94^∗^^##^	1.33 ± 0.44	25.88 ± 4.30^∗∗^^##^

HA	RCP	25.88 ± 1.90	1.61 ± 0.66	0.83 ± 0.26	16.07 ± 4.54
	HCP	22.43 ± 9.68	3.37 ± 1.49^∗^^##^	2.00 ± 0.89^∗^^#^	10.58 ± 6.86
	RCD	27.15 ± 4.43	0.88 ± 0.00^∗^	0.5 ± 0.00	14.75 ± 5.80
	HCD	26.23 ± 6.73	4.45 ± 1.13^∗∗^^##^	1.33 ± 0.44	10.81 ± 1.46

BAC	RCP	6.74 ± 0.68	1.12 ± 0.18	0.5 ± 0.00	4.44 ± 0.72
	HCP	2.97 ± 0.60^∗∗^	0.58 ± 0.17	0.83 ± 0.29	1.79 ± 0.25^∗∗^
	RCD	—	—	—	—
	HCD	—	—	—	—

BMA	RCP	42.34 ± 8.49	0.97 ± 0.2	0.83 ± 0.52	27.86 ± 6.96
	HCP	17.28 ± 5.36^∗∗^^#^	4.68 ± 0.96^∗∗^	2.00 ± 0.65^∗^	7.67 ± 0.89^∗∗^
	RCD	29.58 ± 4.91	6.65 ± 1.98^∗∗^	1.5 ± 0.00	10.25 ± 0.94^∗^
	HCD	15.29 ± 4.61^∗∗^^#^	1.8 ± 0.00^∗^^##^	2.67 ± 0.53^∗^	6.38 ± 2.34^∗∗^

BHA	RCP	16.76 ± 2.12	0.85 ± 0.30	1.50 ± 0.13	9.93 ± 1.83
	HCP	3.54 ± 0.55^∗∗^	1.33 ± 0.58	1.00 ± 0.55	3.16 ± 0.79^∗∗^
	RCD	—	—	—	—
	HCD	—	—	—	—

Mean ± S.D., *n* = 6. ^∗^*p* < 0.05 and ^∗∗^*p* < 0.01 vs. RCP group; ^#^*p* < 0.05 and ^##^*p* < 0.01 vs. RCD group.

## Data Availability

The data used to support the findings of this study are available from the first author (zmr6822@163.com) upon request.
